# Evaluating L1CAM expression in human endometrial cancer using qRT-PCR

**DOI:** 10.18632/oncotarget.9574

**Published:** 2016-05-24

**Authors:** Sara Notaro, Daniel Reimer, Michaela Duggan-Peer, Heidi Fiegl, Annamarie Wiedermair, Julia Rössler, Peter Altevogt, Christian Marth, Alain Gustave Zeimet

**Affiliations:** ^1^ Department of Gynecology and Obstetrics, Medical University of Innsbruck, Innsbruck, Austria; ^2^ Department of Gynecology and Obstetrics, University of Brescia, Brescia, Italy; ^3^ Skin Cancer Unit, German Cancer Research Center (DKFZ), Heidelberg, Germany; ^4^ Department of Dermatology, Venereology and Allergology, University Medical Center Mannheim, Ruprecht-Karl University of Heidelberg, Mannheim, Germany

**Keywords:** L1CAM, endometrial cancer, qRT-PCR, outcome, methylation

## Abstract

**Background:**

Management of endometrial carcinoma (EC) still needs improvement of risk assessment. Recently, L1CAM immunohistochemical (IHC) evaluation showed a unique value to predict the outcome of early EC. However IHC results are often conflicting for lack of inter-laboratory standardisation.

**Methods:**

Here, as a proof of concept and to increase reproducibility we assayed eighty-two EC and 26 normal endometrium samples for L1CAM expression (L1CAM^EXP^) via qRT-PCR. The IHC evaluation was performed in 50 cancer samples. Moreover, we aimed to substantiate the *in-vitro* findings of L1CAM regulation through its promoter methylation (L1CAM^MET^), miR-34a expression and miR-34a promoter methylation. DNA methylation was assessed with MethyLight PCR technique.

**Results:**

High overall concordant results between IHC and RT-PCR evaluations were found. L1CAM^EXP^ was detected in 11% of cancer specimens. These positive cancers exhibited a worse DFS (p=0.032) and OS (p=0.016) in a multivariate COX-regression model. L1CAM^EXP^ predicted distant failure (p=0.007) and L1CAM^MET^ predicted risk-reduction of lymph-node involvement (p=0.005). Inverse correlations between L1CAM^EXP^ and L1CAM^MET^ (p=0.004) and between L1CAM^EXP^ and miR-34a expression (p=0.002) were found.

**Conclusions:**

In conclusion qRT-PCR analysis is a reliable approach to evaluate L1CAM status in EC and L1CAM^EXP^ was highly predictive for distant failure and poor outcome, confirming the large IHC-based studies. Interestingly, L1CAM^MET^ was able to assess the risk of pelvic lymph-node involvement. Especially the latter finding has to be confirmed in larger prospective series.

## INTRODUCTION

Endometrial cancer (EC) represent the sixth most common cancer in women [1]. EC is known to be a good prognosis cancer, since it is usually detected in its early stages and therefore survival rates are around 85% at 5 years [2]. The current challenge is to identify patients with high risk for recurrence avoiding to the others an overtreatment with its associated toxicities. At present clinico-pathological risk factors categorize specific risk classes but nevertheless, local and distant recurrences occur even in low and intermediate risk patients. In order to properly select patients candidate for adjuvant systemic treatment, it is necessary to improve the current risk assessment by the use of highly reliable biomarkers. Recently, several reports showed that immunohistochemical (IHC) detection of L1CAM in endometrial tumor samples is able to discriminate a subset of highly aggressive tumors with adverse clinical outcome [3–6] and high risk of distant recurrences [5,6]. One smaller study showed that only in diabetic patients L1CAM was predictive for lymph node involvement [7].

L1CAM, has been extensively investigated in the last 15 years in relation to its capacity in enhancing cell motility and thereby promoting invasiveness [8] [9] [10] in a variety of human cancers [4,11,12]. Moreover, L1CAM has been linked to EMT (epithelial to mesenchymal transition) in several different cancer types [9], including ECs [3]. EMT is an embryological process characterized by alterations in morphology, cellular architecture, signalling and adhesion leading to a migratory phenotype [13]. There is an increasing body of evidence that tumor spread requests epithelial tumor cells to undergo EMT [14,15].

The control system of L1CAM expression in cancers is complex and is affected by both transcriptional and epigenetic mechanisms [PMID: 26111503]. In the context of EMT up-regulation of L1CAM has been reported to be induced by β-catenin and SLUG [9,16,17]. Down-regulation of L1CAM was found to be driven by the androgen receptor [18], neural restrictive silencer factor/ RE1 silencing transcription factor (NRSF/REST) [19] and PAX-2/8 [20]. Importantly, in endometrial carcinoma cell lines L1CAM has also been shown to be inhibited by DNA methylation of its promoter [21] and by various miRNAs whereby the most reliable data exist for miR-34a [22].

For the best of our knowledge this pilot study for the first time analyses L1CAM expression by RT-PCR in a training set of 82 endometrial carcinoma samples. A direct comparison between IHC and RT-PCR evaluations of L1CAM was performed. Moreover, to substantiate the *in vitro* findings of the regulation of L1CAM expression we analysed also for the first time L1CAM promoter methylation, miR-34a expression and miR-34a promotor methylation in endometrial carcinoma tissue samples.

## RESULTS

### L1CAM expression

**Figure 1:**
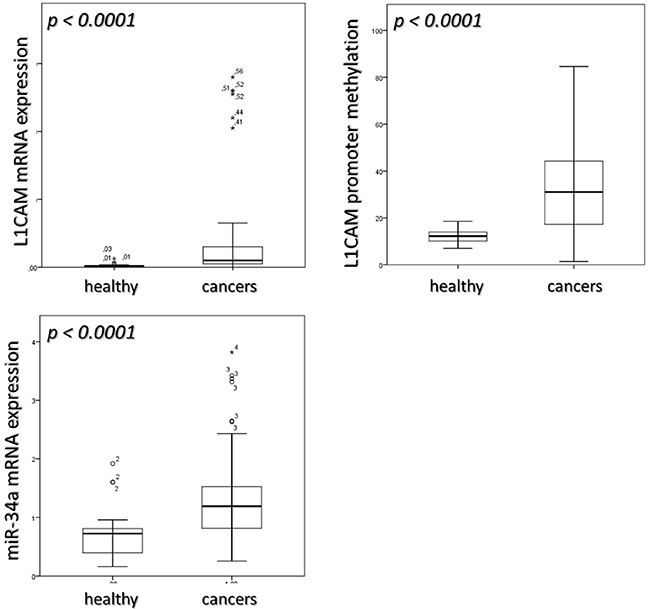
median value of L1CAM expression, its promoter methylation and miR-34a expression in healthy and cancers sample Mann-Whitney test was applied to calculate differences in the two groups.

When compared with normal endometrial tissue L1CAM mRNA levels were significantly higher in endometrial cancers (p< 0.0001, Table 1, Figure 1). Table 2 depicts L1CAM mRNA expression of the endometrial cancers in relation to the classic clinico-pathological characteristics.

**Table 1:** L1CAM expression, its promoter methylation and expression of miR-34a expression in cancers and normal tissues

		25^th^ centile	50^th^ centile	75^th^ centile
*L1CAM expression* ^§^	normal	0.0019	0.0033	0.005
	cancers	0.010	0.020	0.060
Difference:			p < 0.0001^*^	
*L1CAM methylation* ^§§^	normal	10.102	12.214	13.966
	cancers	17.264	31.0535	44.265
Difference:			p < 0.0001^*^	
*miR-34a expression* ^§^	normal	0.398	0.724	0.8094
	cancers	0.724	1.190	1.527
Difference:			p < 0.0001^*^	

* Mann-Whitney test

§ arbitrary units normalized to TBP.

§§ PMR value

**Table 2:** Clinico-pathological characteristics with L1CAM expression and its promotor methylation

		n (%)		*L1CAM expression* ^§^		*L1CAM promoter methylation* ^§§^	
				Median	p-value^#^	Median	p-value^#^
Age	< 69 years^a^	41 (50)	< 69 years	0.02		31.24	
	> 69 years	41 (50)	> 69 years	0.02	0.771	28.53	0.495
Figo stage	Ia	26 (31.7)	I-II	0.02		33.36	
	Ib	40 (48.7)					
	II	4 (4.8)					
	IIIc	12 (14.6)	IIIc	0.03	0.373	12.18	0.009^**^
Risk assessment^b^	low risk	21 (25.6)	low risk	0.01		44.31	
	Intermediate-high risk	59 (71.9)	Intermediate-high risk	0.02	0.003^**^	29.99	0.012^*^
Grading	G1	15 (18.2)	(G1-2)	0.02		32.37	
	G2	42 (51.2)					
	G3	25 (30.4)	(G3)	0.03	0.131	23.66	0.05^*^
Histology	endometrioid	79 (96.3)	endometrioid	0.02		31.05	
	serous	1 (1.2)	non-endometroid	0.06	0.155	21.7	0.553
	clear cell	2 (2.4)					
Myometrial infiltration	< 50%	29 (35.4)	< 50%	0.01		30.86	
	> 50 %^c^	47 (57.3)	> 50 %	0.02	0.127	32.2	0.313
Pelvic nodes metastasis^d^	no	19 (63.3)	No	0.02		35.82	
	yes	11 (36.7)	Yes	0.03	0.679	12.18	0.022^*^

a) Median value in cancer cohort

b) two patients missing

c) six patients missing

d) Lymphadenectomy performed in 30/82 (36.5%)

# Mann-Whitney test

§ Arbitrary units normalized to TBP.

§§ PMR value.

* Significant at the 0.05 level (2-tailed).

** Significant at the 0.01 level (2-tailed).

**Figure 2:**
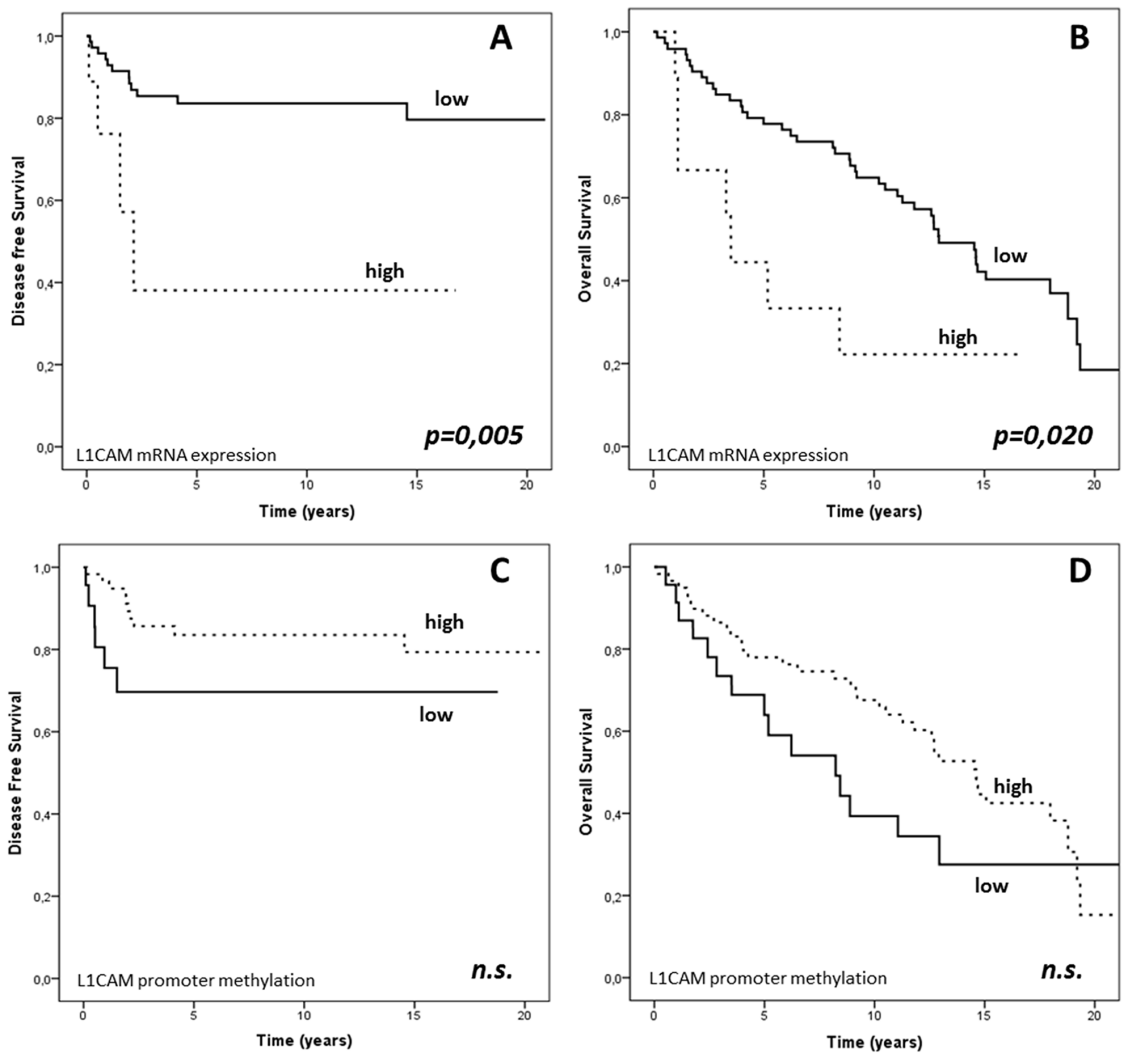
PFS and OS based on L1CAM expression and L1CAM promoter methylation in the endometrial cancer cohort Kaplan-Mayer curves and log-rank test: PFS and OS in the cancer cohort dichotomized according to **A–B.** 89^th^ percentile of L1CAM expression and **C–D.** 29^th^ percentile of L1CAM promoter methylation. Units: § L1CAM mRNA expression: arbitrary units normalized to TBP. §§ L1CAM promoter DNA methylation: PMR values.

In the large majority of the endometrial cancer samples, L1CAM expression measured by RT-PCR was either absent or very weak. An obvious and pronounced increment in the L1CAM expression was observed in nine cases (89^th^ percentile). This let us to set the arbitrary threshold at 0.41 to distinguish between L1CAM positivity and negativity (Supplementary Table S1). Of special note is that L1CAM mRNA expression was found to be significantly higher in intermediate-high risk (median value 0.02; Q_1_-Q_3_: 0.01-0.08) as compared to low risk cancers (median value: 0.01; Q_1_-Q_3_: 0.01-0.02; p=0.003). In the univariate survival analysis the 11% L1CAM positive cancers exhibited an unfavourable DFS (median value 1.21 (Q_1_-Q_3_ 0.50-2.13) years VS 8.63 (Q_1_-Q_3_ 2.48-14.62) years; p=0.005) and OS (median value 3.51 (Q_1_-Q_3_ 1.11-8.43) years VS 12.12 (Q_1_-Q_3_ 5.83-16.06) years p=0.020); (Figure 2A and 2B). Furthermore, in multivariate analysis the independency of L1CAM positivity as a predictor of poor clinical outcome was confirmed for both DFS (HR= 3.60, p=0.037) and OS (HR=2.86, p=0.012); (Table 3).

**Table 3:** Multivariate survival analysis for DFS and OS based on L1CAM expression^†^

	DFS			OS		
	HR	CI95%	p-value	HR	CI95%	p-value
**L1CAM expression ^§^**						
negative	1			1		
positive	3.60	1.08-12.01	0.037^*^	2.86	1.25-6.51	0.012^*^
**FIGO stage**						
I-II	1			1		
III-IV	4.40	1.51-12.84	0.007^**^	1.03	0.42-2.50	0.947
**Grading (G1-2 VS G3)**						
G1-G2	1			1		
G3	1.38	0.41-4.61	0.595	0.31	0.33-1.42	0.399
**Age (median)**						
< 68 years	1			1		
> 68 years	1.42	0.44-4.60	0.549	3.69	2.01-6.76	<0.0001^**^

† Cox-regression.

* significant at the 0.05 level (2-tailed).

** significant at the 0.01 level (2-tailed).

§ arbitrary units normalized to TBP.

§§ PMR value.

**Figure 3:**
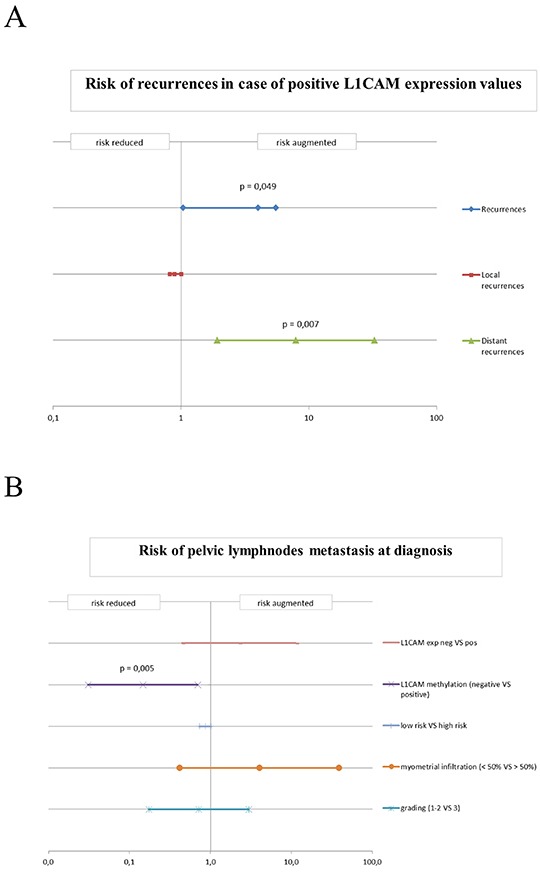
**A.** Risk of recurrences in case of positivity for L1CAM expression in the endometrial cancer cohort. Odds ratio (OR) for the probability of experiencing a recurrence (both local and distant) for positive values of L1CAM. **B.** Risk of pelvic lymph node metastasis at diagnosis. Odds ratio (OR) for expression of L1CAM, methylation of L1CAM, risk assessment, myometrial infiltration and high-low grade cancers. The bars indicate the value of the odds ratio and the confidence intervals at 95%.

The odds ratio for experiencing a recurrence (either loco-regional or distant) in case of L1CAM positivity was 4.07 (0.951-17.39 p=0.045) and the risk for distant failure was 6.5 (1.442 − 29.305, p=0.010). However, L1CAM expression failed to be a powerful predictor for loco-regional recurrence (Figure 3A).

Of special note was that in the investigated cohort of patients L1CAM expression was unable to predict the risk for lymph node involvement (Figure 3B).

### L1CAM mRNA expression and IHC staining

L1CAM IHC staining was performed for 50 patients randomly selected in the entire cohort of 82 patients.

Comparison between semiquantitative immunohistochemical evaluation and RT–PCR quantification of L1CAM transcripts resulted in a highly significant correlation (p < 0.0001; r_s_ =0.663). In Table 4 the overall concordance and discordance of the results of both methods in L1CAM determination are listed. It is noteworthy that there is a very high rate of concordance regarding the negative cases but a limited agreement in L1CAM positive cancers. When IHC was considered “standard”, sensitivity and specificity of qRT-PCR for detection of L1CAM were 40% and 100%, respectively. Cohen Kappa value resulted 0.516; p= 0.003.

**Table 4:** Correlation of L1CAM qRT-PCR and IHC

		PCR		
		positive	negative	tot.
	IHC positive	4	6	10
	IHC negative	0	40	40
tot.		4	46	50

In 12 borderline cases of RT-PCR positivity (from 0.04 to 0.14), IHC evaluation presented six negative cases and six positive cases. None of the tumors which showed borderline IHC detection (less than 10%) revealed to be RT-PCR positive.

### Methylation of the L1CAM promoter

The methylation of the L1CAM promoter was also significantly higher in cancers (median value: 31.05; Q_1_-Q_3_: 17.26-44-26) as compared to healthy endometrial tissue (median value 12.21; Q_1_-Q_3_: 10.10-13.96; p < 0.0001). Table 1, Figure 1.

The relations of methylation of the L1CAM promoter with the classic clinico-pathological characteristics are given in Table 2. Noteworthy, L1CAM promoter methylation was found to be significantly lower in FIGO stage IIIc (median value: 12.18; Q_1_-Q_3_: 3.83-28.52) compared to stage I-II cancers (median value 33.35; Q_1_-Q_3_:21.58-44.31; p = 0.009), in grade 3 (median value: 23.66; Q_1_-Q_3_: 9.61-35.57) compared to grade 1 and 2 cancers (median value: 32.37; Q_1_-Q_3_: 21.58-44.31; p = 0.05) and in cancers with metastatic lymph nodes (median value: 12.18; Q_1_-Q_3_: 3.83-28.52) in comparison with negative lymph node status nodes (median value: 35.81; Q_1_-Q_3_: 21.70-45.77; p = 0.022). As no data at all are available on clinical relevance of L1CAM promoter methylation we calculated an optimal cut-off point by stratifying patients of this training set into 2 groups according to their L1CAM promoter methylation level, using cut-off points set arbitrarily between the 20^th^ and 80^th^ percentile. Survival curves were calculated for each of these cut-offs, and p values are calculated. Although there was no cut-off resulting in significant differences in survival, the optimal threshold value with the best non-significant clinical outcome was obtained for the 28^th^ percentile (19.7) which was then used as cut off between L1CAM positive and negative methylation status. In univariate analysis high promoter methylation of L1CAM resulted in a non-significant better DFS (median value: 9.10; Q_1_-Q_3_: 2.76-16.05 years VS 2.14; Q_1_-Q_3_: 0.51-11.25 years; p= 0.096) and OS (median value: 12.71; Q_1_-Q_3_: 5.90-16.52 years VS 7.23; Q_1_-Q_3_: 2.63-12.80 years; p = 0,115) (Figure 2).

When this threshold was applied to predict lymph node involvement a positive methylation status of L1CAM promoter revealed to be significant predictor of a reduced risk for lymph nodes metastasis (odds ratio = 0.100; [0.02-0.52]; assailed with a positive predictive value (PPV) of 72.6 % and negative predictive value (NPV) of 79 %; p = 0.005). None of the other classical clinico-pathological parameters revealed to be a better predictor for lymph node involvement, this includes also the multifactor risk assessment which in our cohort only showed a predictive trend with an odds ratio of 0.864 (0.732-1.020, p=0.096) for lymph node spread (Figure 3B).

### miR-34a expression and promoter methylation

miR-34a expression revealed to be significantly higher in cancers (median value: 1.19; Q_1_-Q_3_: 0.81-1.52) than in healthy endometrial tissue (median value: 0.72; Q_1_-Q_3_: 0.39-0.80; p < 0.0001) Table 1, Figure 1. However, no differences between normal endometrium and endometrial cancers have been found regarding the specific methylation of the miR-34a promotor (Table 1), which was absent in healthy samples and low but existent in five (6 %) of the cancers. Four out of these five patients were found to bear high risk endometrial cancers. The assignment to the high risk type of tumors was mostly based on poor differentiation. Nonetheless, these five patients did not exhibit an obvious inferior clinical course compared to patients without methylation of miR-34a promoter.

Neither miR-34a expression nor miR-34a promoter methylation did demonstrate any prognostic relevance in the herein examined cohort of patients with endometrial cancer.

### Inverse correlations

Regarding regulation of L1CAM expression, an inverse correlation between L1CAM mRNA levels and methylation of its promoter was revealed (r_s_: − 0,318; p=0.004). Moreover, L1CAM mRNA expression correlated also negatively with miR-34a expression (r_s_: − 0.343; p=0.002). These associations have been exclusively found in cancers but not in normal endometrium tissue (Table 5).

**Table 5:** correlations between L1CAM, L1CAM methylation, miR-34a and miR-34a methylation

			L1CAM promoter methylation	miR-34a expression	miR-34a promoter methylation
Spearman's rho	L1CAM expression	Correlation Coefficient	−.318^**^	−.343^**^	.073
		Sig. (2-tailed)	***.004***	***.002***	.517
		N	82	81	82
	L1CAM promoter methylation	Correlation Coefficient		.124	.084
		Sig. (2-tailed)		.269	.451
		N		81	82
	miR-34a expression	Correlation Coefficient			−.102
		Sig. (2-tailed)			.366
		N			81

s** Correlation is significant at the 0.01 level (2-tailed).

### L1CAM regulation

In order to elucidate the regulatory potency of both inhibitory systems we categorized patients in negative and positive for miR-34a expression (25^th^ percentile as cut off level) and for L1CAM promoter methylation (cut off set at 29^th^ percentile) as depicted in Supplementary Table S2. Endometrial cancers rated negative for both inhibitory mechanisms presented with higher L1CAM values as compared to tumors which showed significant inhibitory activity in both systems (p = 0.011). However, when the regulatory mechanism were tested with regard to their influence on clinical outcome, survival curves (Supplementary Figure S1) exhibited that methylation of L1CAM promoter appears to have a more pronounced effect on favourable clinical impact than miR-34a levels did.

## DISCUSSION

By the best of our knowledge, this is the first study evaluating the L1CAM status in endometrial cancer on the transcriptome level using qRT-PCR on fresh frozen tissue samples. We were motivated to perform this investigation by means of a reliable and well-established quantitative technique as in various IHC studies a considerable range of different L1CAM positivity rates have been reported in endometrial cancer [3] [7]. This range varying from 7% to 29% was only partly explicable by different thresholds used and the portion of serous and clear cell cancers included in the various analyses. [3–7]. Although qRT-PCR enables an objective evaluation of L1CAM mRNA levels, this method however, has the disadvantage not to reflect the protein level of L1CAM and thus post-transcriptional regulations that have been descripted by Schirmer et al. and Doberstein et al., are not taken into consideration [22,23]. Nonetheless, we found a highly significant correlation between qRT-CR and IHC with a high rate of concordance in the L1CAM negative samples but with noteworthy discordant results in positive cancers. In fact, whole tissue RT-PCR may underestimate L1CAM positivity because it may be unable to adequately assess unevenly distributed L1CAM expressing small cell clusters, which have been frequently shown with IHC in endometrial cancer and were found to be of clinical relevance. Although, in the present approach L1CAM expression was revealed to be significantly higher in cancer samples as compared with healthy endometrium, the large majority of endometrial cancers proved either to lack L1CAM expression at all or to exhibit only very weak L1CAM expression. The resulting positivity rate of 11% was considerably lower compared to the 17% determined with immunohistochemistry (≥ 10% L1CAM expressing cells) in our multicenter series of 1021 early type 1 endometrial cancers [5] and was closer to the reported positivity rate of 7% revealed for the retrospective analyses obtained from the PORTEC-1 and PORTEC-2 data [6]. It should nonetheless be emphasized, that in both mentioned previous studies only stage I cancers have been included where in the present investigation 14.6 % cancer of FIGO stage IIIc cancers with positive lymph nodes have been included. Furthermore, it should be considered that cancers rated high risk by multifactor assessment, known to have a higher likelihood to be L1CAM positive, are overrepresented with 73 % in this cohort. The issue of the positive rate of L1CAM in endometrial cancer is of great importance to allow adequate cohort estimations when L1CAM-based clinical studies are planned in future.

In accordance to recent IHC reports, L1CAM positive cancers identified by RT-PCR exhibited a significant worse disease-free (DFS) and overall survival (OS). Independency of these findings was confirmed in multivariate Cox regression for both DFS and OS. Furthermore, elevated L1CAM mRNA levels were highly associated with distant recurrence of endometrial cancer. All these findings are in agreement with the data of both large IHC studies [5,6]. Nonetheless, regarding loco-regional failure no significant association with L1CAM mRNA expression could be revealed in the herein examined series. This is in accordance with the IHC results of Bosse et al. but disagrees with our large retrospective IHC evaluation, where L1CAM positivity was also highly predictive for locoregional recurrence [5].

As the most consistent outcome throughout the various studies is the association of L1CAM positivity with distant relapse, it appears reasonable that patients with L1CAM positive cancers are candidates for a systemic adjuvant chemotherapy. However, so far there are no clinical data available on chemosensitivity of L1CAM positive endometrial cancers. *In vitro* investigations in pancreatic cancer cells have shown that L1CAM expression is rather associated with resistance to conventional cytotoxic agents [24]. Therefore a systemic treatment with a humanized anti-L1CAM antibody, which is currently under investigation, should be considered as an alternative treatment option in endometrial cancer [25].

A further goal of this study was to investigate the postulated main inhibitory regulation systems of L1CAM expression, namely L1CAM promoter methylation and miR-34a expression together with its own specific promoter methylation. Indeed, in our cohort of patients we found a highly negative correlation between L1CAM expression and the methylation of its promotor as well as between L1CAM and miR-34a expression. These results strongly corroborate the recently reported *in vitro* findings obtained on endometrial cancer cell lines [26].

Regarding miR-34a we were astonished to see firstly a more abundant expression in the cancer samples as compared to healthy endometrium and secondly a complete lack of methylation of the miR-34a promotor in normal endometrial tissue. This tempts us to speculate that normal endometrium is apparently a tissue with very low constitutive miR-34a expression which does not appear to be inhibited by miR-34a promoter methylation.

However the main finding when investigating the regulation of L1CAM was that presence of specific methylation at the L1CAM promotor could be predictive for a negative lymph node status before primary surgery. Due to the small sample size of stage IIIc endometrial cancers in our cohort, these findings can only be regarded as hypothesis generating, the predictive odds ratio of 0.100 proved to be very impressive. In our evaluation, none of the traditional risk factors including the multifactor risk assessment for the presence of lymph-nodes metastasis was superior to methylation of L1CAM promotor. Due to the limited sample size, these findings so far do not allow general conclusions but should be the inciting basis for larger evaluations on this issue. Furthermore, to date, these results may be of limited practical relevance as MethyLight technique may not be sufficiently widespread and adequately established to allow routine determination of L1CAM promotor methylation prior surgery. Furthermore, the herein presented preliminary data have first to be confirmed in a larger series and especially validated for curettage material.

The most relevant weakness of the present study is related to the retrospective character of our evaluation. Furthermore, in this pilot trial patients with FIGO IIIc were included and therefore comparisons with the outcome of earlier IHC studies may be of limited value. However, the inclusion of those cases, which all were considered to be high risk FIGO stage I cancers during surgery and have been up-staged due to microscopically positive lymph nodes, was a prerequisite to study the issue of prediction of pelvic lymph node involvement by L1CAM and miR-34a expression as well as the methylation of their respective promotors.

In conclusion, we herein present a proof of concept to determine L1CAM status on the transcriptome level and to extend the number of reliable methods for L1CAM determinations in endometrial cancers. With regard to the clinical outcome of patients, RT-PCR was comparable to IHC and the L1CAM positive rate of cancers revealed with RT-PCR was in between the positive rates of both so far most important IHC studies. Of special note is the finding that strong promotor methylation of L1CAM proved to predict non-involvement of pelvic lymph nodes and could potentially represents a reliable biomarker for preoperative estimation of lymph node involvement.

## MATERIALS AND METHODS

### Patients

We investigated 82 patients with endometrial carcinomas who underwent primary surgery between 1995 and 2005 at the Department of Obstetrics and Gynecology, Innsbruck Medical Hospital, Austria. We also investigated control samples of healthy endometrium from 24 patients undergoing hysterectomy for non-malignant reasons. We did not exclude patients on the basis of age. Cases for this pilot study were randomly selected on the basis of availability of tissues and have not been stratified for known preoperative or pathological prognostic factors. The clinical stage of tumours was assessed according to the FIGO staging system 2008 [27]. Histological types and grades of tumours were determined by WHO criteria. The survival time and follow-up period was calculated from the date of surgery. The median follow-up period was 11.6 years (range 0.17–21.88). After primary treatment, all the patients were monitored by our department at intervals increasing from 3 months to 1 year until death or the end of the study. Clinico-pathological characteristics are resumed in Table 2. The vast majority (85.3 %) of the included patients were in FIGO stage I and II. However, in order to explore the predictive value for lymph-node involvement of the investigated biologic markers, also FIGO stage IIIc endometrial cancers were included in the current study. Cancers were divided in low risk (myometrial invasion <50%, grade I and II, no lymph space or vascular invasion) and intermediate- high risk (myometrial invasion <50% and grade III, myometrial invasion ≥50% and any grade, lymph space or vascular invasion or clear cell/serous histology). Time from surgery to last follow-up or until death from any cause is defined as overall survival (OS) and time from diagnosis until recurrence of tumor or death from any cause was defined disease free survival (DFS). Loco-regional recurrences are defined as failures in the lower pelvis, distant recurrences are referred to all recurrences of other localization. Follow up information was available for all the patients. Written informed consent regarding tissue and data use for scientific purposes was obtained from all participating patients. Data use for statistical analyses was done in a pseudo-anonymized manner. The retrospective study was approved by the local ethics committee. All studies were conducted according to the ethical principles suggested in the Declaration of Helsinki.

### mRNA and miRNA expression analysis

Tumor specimens were obtained immediately after surgery and brought to our pathologist. A part of the tissue was pulverized under cooling with liquid nitrogen and stored at −80°C. Total cellular RNA extraction and reverse transcription of RNA were performed as recently described [28]. Primers and probes for the TATA box-binding protein (TBP; a component of the DNA-binding protein complex TFIID as an endogenous RNA control) were used according to Bieche et al (2001). [29] Primers and probes for L1CAM were determined with the assistance of the computer program Primer Express (Life Technologies, Carlsbad, CA, USA). BLASTN searches were conducted to confirm the total gene specificity of the nucleotide sequences chosen for the primers and probes. To prevent amplification of contaminating genomic DNA, the probe was placed at the junction between two exons. L1CAM Forward primer: 5′-TTC GTC CTG AAG CAC TGT TGT C-3′; L1CAM Reverse-primer: 5′-GGA GCG CCT GTG CCC-3′; L1CAM TaqMan probe: 5′-FAM-ATC CTC GTC CAG CCA CTG AAC A-3′-TAMRA. PCR reactions were performed as recently described [27]. A TaqMan microRNA assay specific for miR-34a (Assay ID 000426) was used to detect and quantify mature miR-34a. miRNA expression was normalized to RNU6B (Assay ID 001093) using the 2^−ΔΔCt^ method. The assays were performed in accordance with manufacturer's instructions (Applied Biosystems, Carlsbad, USA) using an ABI Prism Detection System.

### Immunohistochemical staining and evaluation

Immunohistochemical staining was performed as previously described [3, 4]. Briefly, 3- to 4-μm thick paraffin sections were cut and mounted on Superfrost Plus slides that were exposed in a pressure cooker to EDTA buffer, pH 8.0, for antigen retrieval. An automated immunohistochemistry procedure was performed using the I6000 immunostainer (Biogenics, San Ramos, CA). Endogenous peroxidase activity was blocked by 10 minutes of treatment with 3% hydrogen peroxide in methanol. Primary L1CAM antibody (clone L1-40.10) was obtained after immunization of mice with human L1-Fc protein comprising the ectodomain of L1CAM [30]. Slides were incubated with primary antibodies for 45 minutes, and immunoperoxidase staining was accomplished using the Supersensitve Detection Kit with AEC or DAB (Zymed Labs, San Francisco, CA) as substrates, then counterstained with hematoxylin before coverslipping and reading by light microscopy. Omission of the primary antibody was used as a negative control and a highly L1CAM-expressing serous ovarian cancer as a positive control. As previously reported, If 10% or more of the tumor cells showed L1CAM staining, the cancer was rated positive. The stained sections were examined by two pathologists blinded for clinical outcome data.

### DNA methylation analysis

Genomic DNA from endometrial tissues was isolated using the DNeasy tissue kit (Qiagen, Hilden, Germany). Bisulfite modification was performed using the EZ DNA Methylation-Gold Kit (Zymo Research, Orange, CA, USA) according to the manufacturer's instructions. MethyLight PCR analysis was done as described previously [31]. The PMR value (percentage of fully methylated reference) was calculated to determine the DNA methylation measurement. Primers and probes for *L1CAM* were determined with the assistance of the computer program Primer Express version 2.0.0 (Applied Biosystems, Foster City, CA, USA) to produce a 68-base-pair PCR amplicon (nucleotide positions c4070008-4069940 as defined by GenBank accession number NT_167198.1; −10,671 nucleotides to −10,603 nucleotides upstream from the transcription start site). An E-box for the binding of Slug/Snail is located within the amplicon (within the forward primer sequence). The amplicon is placed in the promoter 1 region [32]. Primers and probes for *MIRN34A* were also determined with the assistance of Primer Express software version 2.0.0 (Applied Biosystems, Foster City, CA, USA) to produce a 111-base-pair PCR amplicon (nucleotide positions 33.932-34.043 as defined by GenBank accession number EF570049; −192 nucleotides to −81 nucleotides upstream from exon 1). Genomic DNA not treated with bisulfite (unmodified) was not amplified with the primers (data not shown). Primer sequences were: *L1CAM* Forward primer: 5′-AAT ACT CCC TTA ACC TCG ACC TAA CC-3′, *L1CAM* Reverse primer: 5′-GGC GTT GCG TGT AGG TGT T-3′, *L1CAM* TQM Probe: 5′FAM-TCG ACG ACG CCG ACC AAC GAT-3′BHQ1 (probe). *MIRN34A* Forward primer 5′-TCC TTC CTA CTC GTA CCA CCA AA-3′, *MIRN34A* Reverse primer 5′-AGG TGG AGG AGA TGT CGT TGT T-3′, *MIRN34A* Taq Man probe: 5′FAM-CGT CTC TCC AAC CCG AAA TCC GAA AAA-3′-BHQ1. CpG islands in the analyzed genes were identified using a CpG island searcher (www.uscnorris.com/cpgislands/cpg.cgi) which screens for CpG islands which meet the criteria and algorithm described by Takai and Jones [33].

### Statistics

The comparison between two continuous variables is made by Mann-Whitney test and between more continuous variable with Kruskal Wallis test. Risk was analysed with odds ratio and chi-square tests to evaluate significant values. The analysis of survival was done with Kaplan Mayer curves and log-rank test. The Cox-regression analysis is used in the multivariate survival analysis. For elimination of variables we applied a backward variable selection procedure. A p-value of 0.1 was used for the exclusion of variables; all the other tests were performed using a 0.05% level of significance. Correlations are performed with Spearmans'Rho test. All the statistical analyses were performed using SPSS v.22.

## SUPPLEMENTARY FIGURE AND TABLES


