# Supplementary material for: Evaluating L1CAM expression in human endometrial cancer using qRT-PCR
Source: Oncotarget. 2016 May 24;7(26):40221–32. doi: 10.18632/oncotarget.9574 (PMC5130004; doi:10.18632/oncotarget.9574)
Supplement: Supplementary file 1 [file oncotarget-07-40221-s001.pdf]

Evaluating L1CAM expression in human endometrial cancer using qRT-PCR

SUPPLEMENTARY FIGURE AND TABLES

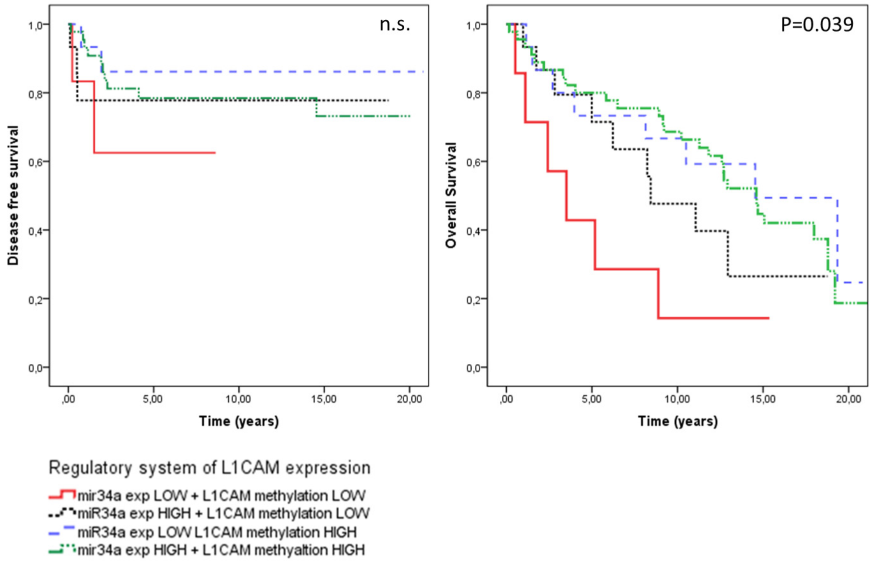

Supplementary Figure S1: Survival curves based on the L1CAM regulation systems. Kaplan Mayer curves for PFS and OS are depicted in four groups on patients which are the follows: negative and positive for miR-34a expression (25th percentile as cut off level) and negative and positive for L1CAM promoter methylation (cut off set at 29th percentile). Log rank test was applied.

Supplementary Table S1: L1CAM vales based on percentiles

Value	Frequency	Percent	Cumulative Percent
.00	5	6.1	6.1
.01	31	37.8	43.9
.02	13	15.9	59.8
.03	5	6.1	65.9
.04	5	6.1	72.0
.05	2	2.4	74.4
.06	3	3.7	78.0
.07	1	1.2	79.3
.08	2	2.4	81.7
.09	1	1.2	82.9
.10	2	2.4	85.4
.12	1	1.2	86.6
.13	2	2.4	89.0
.41	1	1.2	90.2
.44	1	1.2	91.5
.51	1	1.2	92.7
.52	2	2.4	95.1
.56	1	1.2	96.3
1.54	1	1.2	97.6
3.49	1	1.2	98.8
4.06	1	1.2	100.0
Total	82	100.0	

The large majority of the endometrial cancer samples L1CAM expression was either absent or very weak.
A pronounced increment in the L1CAM expression was observed in nine cases (89th percentile), This let us to set the arbitrary threshold at 0.41 to distinguish between L1CAM positivity and negativity.

Supplementary Table S2: Inhibiting mechanism of L1CAM expression (miR-34a expression and L1CAM promoter methylation) grouped with regard to their respective activity (high or low)

miR-34a expression	L1CAM methylation	n.	L1CAM expression (median) §	p-value*
low	low	7	0.06	
low	high	15	0.04	*0.011*
high	low	15	0.04	
high	high	45	0.01	

Median value of L1CAM expression are given for the form possible constellations. Cut off for miR-34a expression: 25th percentile, for L1CAM methylation: 29th percentile.
§ arbitrary units normalized to TBP.
* Kruskal Wallis test.
